# Supplementary figures and images for: A small molecule targeting protein translation does not rescue spatial learning and memory deficits in the hAPP-J20 mouse model of Alzheimer’s disease
Source: PeerJ. 2016 Oct 19;4:e2565. doi: 10.7717/peerj.2565 (PMC5075699; doi:10.7717/peerj.2565)

A

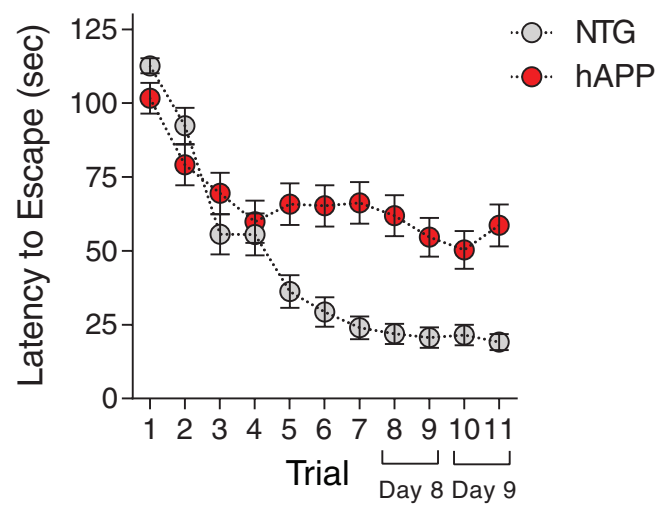

B

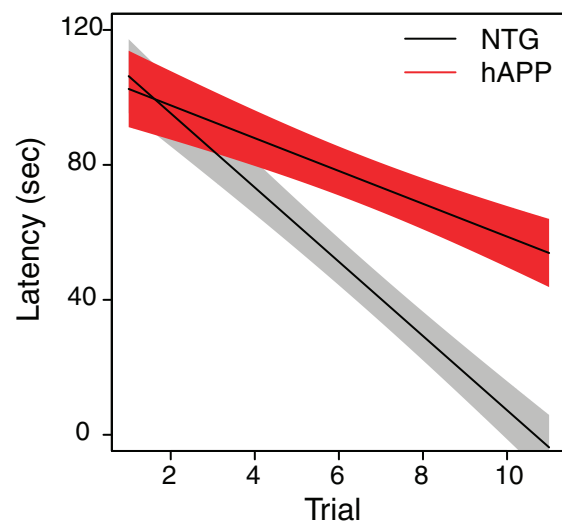

Supplement: Figure S1 — (A, B) J20 (N = 45) and nontransgenic (NTG) (N = 48) mice were trained in the Morris water maze (MWM) over 11 trials (A). (B) Learning rates to locate the hidden platform were analyzed using a linear mixed model. J20 mice showed an impairment to locate the hidden platform compared to NTG mice (linear mixed effects regression model, NTG learning rate = 11 sec/day (5–95% CI [12.5–9.5] sec/day), J20 learning rate = 4.9 sec/day (5–95% CI [7.0–2.7] sec/day, p = 2.1x10^−8^). CI, confidence interval. [file peerj-04-2565-s002.pdf]

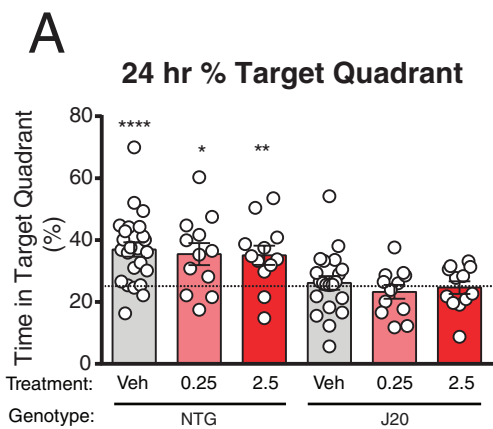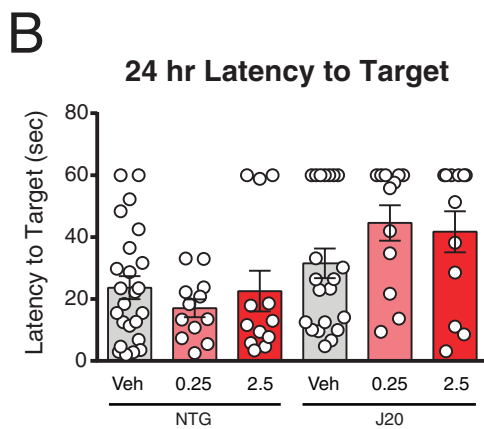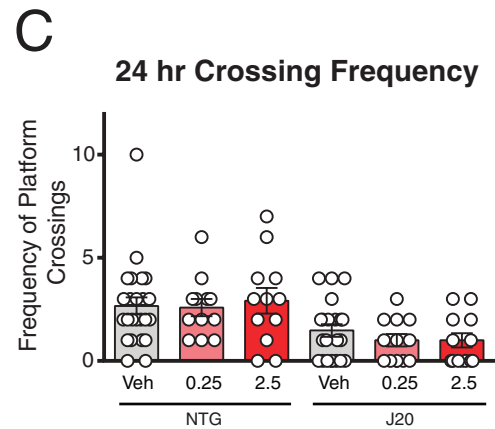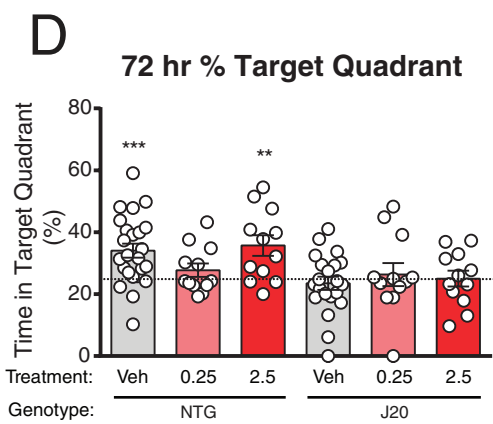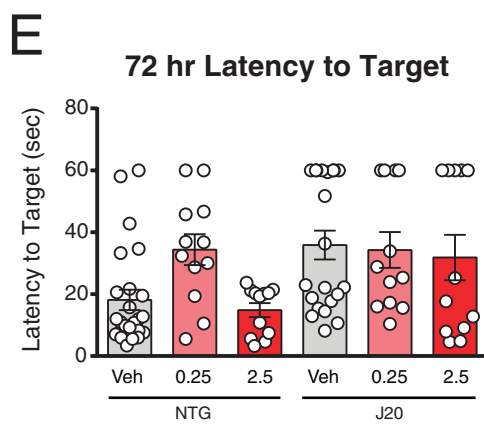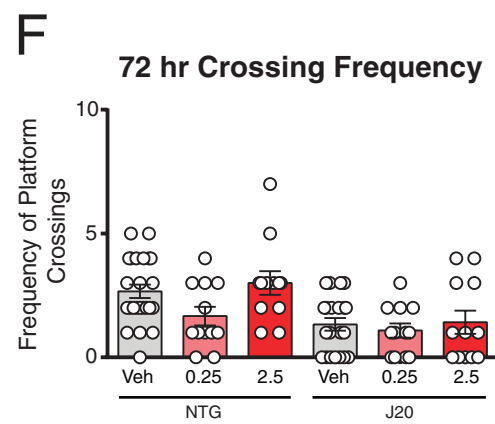

Supplement: Figure S2 — (A-F) Probe data shown in Figure 4E-J was reanalyzed by pooling vehicle control nontransgenic (NTG) and hAPP-J20 (J20) groups to increase statistical power. (A–C) 24-hr probe trial. (A) NTG mice spent more time in the target quadrant than would be expected by chance, while J20 mice did not (one sample t test against 25% (dotted line): NTG vehicle p < 0.0001, NTG ISRIB 0.25 mpk p = 0.01, NTG ISRIB 2.5 mpk p = 0.008, J20 vehicle p = 0.61, J20 ISRIB 0.25 mpk p = 0.45, J20 ISRIB 2.5 mpk p = 0.87). Treatment with ISRIB did not increase the percentage of time spent in the target quadrant (two-way ANOVA: treatment F(2,87) = 0.42, p = 0.66; genotype F(1,87) = 25.10, p < 0.0001; treatment × genotype F(2,87) = 0.06, p = 0.95). (B) Treatment with ISRIB did not reduce the latency to first cross the target platform location (two-way ANOVA: treatment F(2,87) = 0.47, p = 0.63; genotype F(1,87) = 17.94, p < 0.0001; treatment × genotype F(2,87) = 2.03, p = 0.14). (C) Treatment with ISRIB did not increase the number of platform crossings (two-way ANOVA: treatment F(2,87) = 0.23, p = 0.80; genotype F(1,87) = 19.16, p < 0.0001; treatment × genotype F(2,87) = 0.40, p = 0.67). (D–F) 72-hr probe trial. (D) At 72 hours after completion of the last training trial, NTG mice continued to spend more time in the target quadrant compared to chance, except for the group treated with 0.25 mpk ISRIB (one sample t test against 25% (dotted line): NTG vehicle p = 0.0006, NTG ISRIB 0.25 mpk p = 0.23, NTG ISRIB 2.5 mpk p = 0.008, J20 vehicle p = 0.49, J20 ISRIB 0.25 mpk p = 0.73, J20 ISRIB 2.5 mpk p = 0.99). Treatment with ISRIB did not increase the percentage of time spent in the target quadrant (two-way ANOVA: treatment F(2,87) = 0.61, p = 0.55; genotype F(1,87) = 11.15, p = 0.001; treatment × genotype F(2,87) = 1.73, p = 0.18). (E) Treatment with ISRIB did not reduce the latency to first cross the target platform location (two-way ANOVA: treatment F(2,87) = 2.17, p = 0.12; genotype F(1,87) = 8.05, p [file peerj-04-2565-s003.pdf]
